# Supplementary material for: A translational program that suppresses metabolism to shield the genome
Source: Nat Commun. 2020 Nov 13;11:5755. doi: 10.1038/s41467-020-19602-2 (PMC7666154; doi:10.1038/s41467-020-19602-2)
Supplement: Supplementary file 1 — Supplementary Information [file 41467_2020_19602_MOESM1_ESM.pdf]

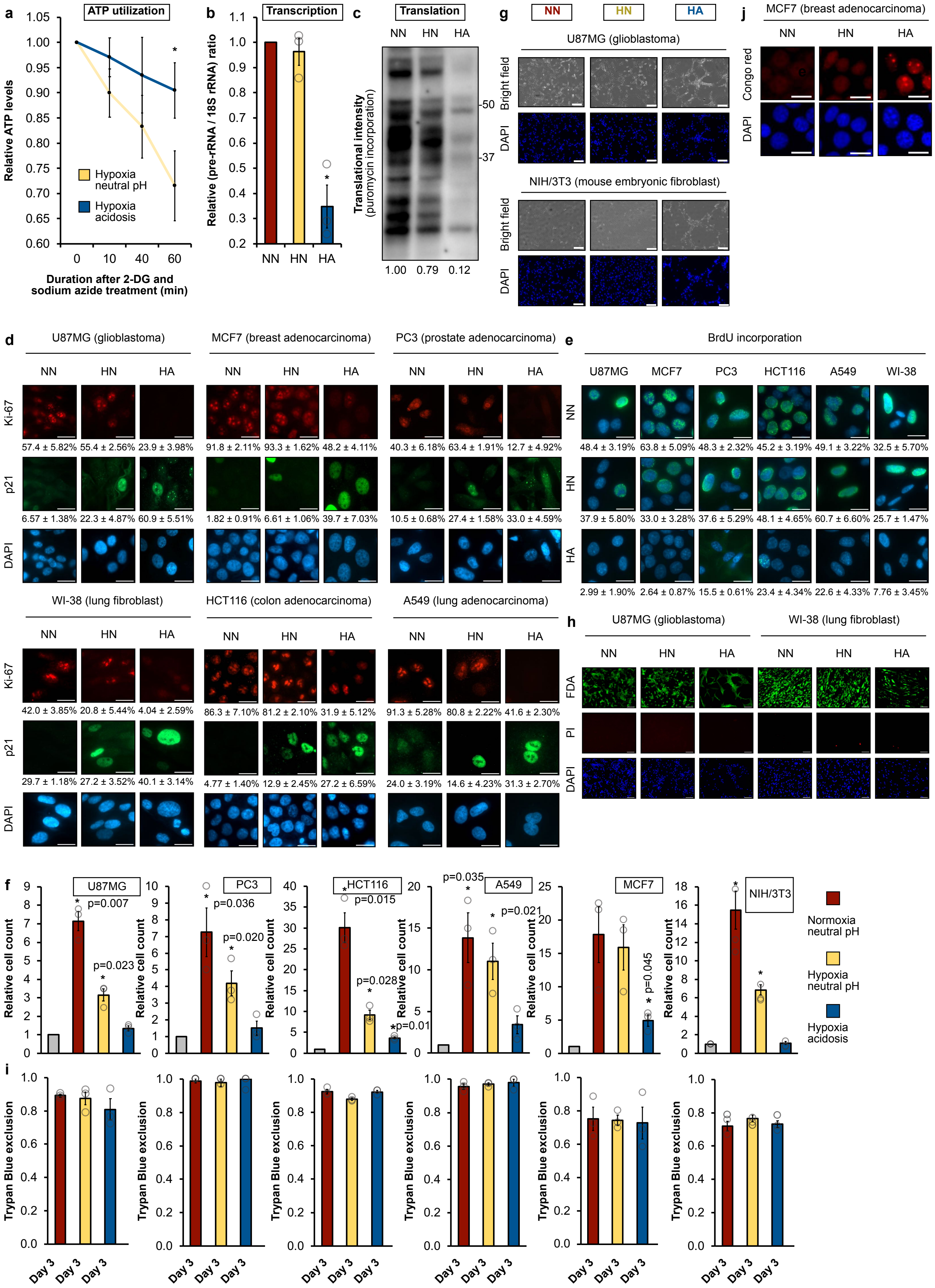

a
ATP utilization
Relative ATP levels
1.05
1.00
0.95
0.90
0.85
0.80
0.75
0.70
0.65
0.60
Hypoxia neutral pH
Hypoxia acidosis
0
10
40
60
Duration after 2-DG and sodium azide treatment (min)
b
Transcription
Relative (pre-rRNA / 18S rRNA) ratio
1.1
1.0
0.9
0.8
0.7
0.6
0.5
0.4
0.3
0.2
NN
HN
HA
c
Translation
NN HN HA
Translational intensity (puromycin incorporation)
-50
-37
1.00 0.79 0.12
g
NN
HN
HA
U87MG (glioblastoma)
Bright field
DAPI
NIH/3T3 (mouse embryonic fibroblast)
Bright field
DAPI
j
MCF7 (breast adenocarcinoma)
NN HN HA
Congo red
DAPI
d
U87MG (glioblastoma)
MCF7 (breast adenocarcinoma)
PC3 (prostate adenocarcinoma)
NN HN HA
Ki-67
57.4 ± 5.82% 55.4 ± 2.56% 23.9 ± 3.98%
91.8 ± 2.11% 93.3 ± 1.62% 48.2 ± 4.11%
40.3 ± 6.18% 63.4 ± 1.91% 12.7 ± 4.92%
p21
6.57 ± 1.38% 22.3 ± 4.87% 60.9 ± 5.51%
1.82 ± 0.91% 6.61 ± 1.06% 39.7 ± 7.03%
10.5 ± 0.68% 27.4 ± 1.58% 33.0 ± 4.59%
DAPI
WI-38 (lung fibroblast)
HCT116 (colon adenocarcinoma)
A549 (lung adenocarcinoma)
Ki-67
42.0 ± 3.85% 20.8 ± 5.44% 4.04 ± 2.59%
86.3 ± 7.10% 81.2 ± 2.10% 31.9 ± 5.12%
91.3 ± 5.28% 80.8 ± 2.22% 41.6 ± 2.30%
p21
29.7 ± 1.18% 27.2 ± 3.52% 40.1 ± 3.14%
4.77 ± 1.40% 12.9 ± 2.45% 27.2 ± 6.59%
24.0 ± 3.19% 14.6 ± 4.23% 31.3 ± 2.70%
DAPI
e
BrdU incorporation
U87MG MCF7 PC3 HCT116 A549 WI-38
NN
48.4 ± 3.19% 63.8 ± 5.09% 48.3 ± 2.32% 45.2 ± 3.19% 49.1 ± 3.22% 32.5 ± 5.70%
HN
37.9 ± 5.80% 33.0 ± 3.28% 37.6 ± 5.29% 48.1 ± 4.65% 60.7 ± 6.60% 25.7 ± 1.47%
HA
2.99 ± 1.90% 2.64 ± 0.87% 15.5 ± 0.61% 23.4 ± 4.34% 22.6 ± 4.33% 7.76 ± 3.45%
h
U87MG (glioblastoma)
WI-38 (lung fibroblast)
NN HN HA NN HN HA
FDA
PI
DAPI
f
U87MG
p=0.007
p=0.023
PC3
p=0.036
p=0.020
HCT116
p=0.015
p=0.028
p=0.01
A549
p=0.035
p=0.021
MCF7
p=0.045
NIH/3T3
Relative cell count
Normoxia neutral pH
Hypoxia neutral pH
Hypoxia acidosis
i
Trypan Blue exclusion
1.0
0.8
0.6
0.4
0.2
0.0
Day 3 Day 3 Day 3

**Supplementary Figure 1**. (a) Measurements of ATP utilization in U87MG subjected to hypoxia neutral pH and hypoxia acidosis conditions. Data represent mean ± SEM (n=3). * indicates p = 0.045 compared to corresponding hypoxia neutral pH condition, two-sided student’s t-test. (b) Relative transcriptional intensity (by rRNA synthesis measurements) in U87MG subjected to the indicated conditions. NN: Normoxia neutral pH; HN: Hypoxia neutral pH; HA: hypoxia acidosis. Data represent mean ± SEM (n=3). * indicates p = 0.017 compared to NN, two-sided student’s t-test. (c) Representative immunoblots of puromycin incorporation (measure of global translational intensity) in U87MG subjected to the indicated conditions. Densitometry represent mean (n=3). Representative images of (d) Ki-67 and p21 immunocytochemistry, (e) BrdU staining, and (f) cell number measurements in various human and mouse cell lines subjected to indicated conditions. Data represent mean ± SEM (n=10 (d, e); n=3 (f)). * indicates p <.05 (exact p value in figure) compared to day 1, two-sided student's t-test. Scale bars: 20 μm. (g) Cell morphology and patterning of U87MG and NIH/3T3 subjected to indicated conditions. Scale bars: 100 μm. Measurements of cell death and viability by (h) fluorescein diacetate (FDA) and propidium iodide (PI) staining and (i) Trypan Blue exclusion in various human and mouse cell lines subjected to indicated conditions. Scale bars: 20 μm. Data represent mean ± SEM (n=5).(j) Representative images of Congo red staining in U87MG subjected to indicated conditions (n=5) Scale bars: 20 μm.

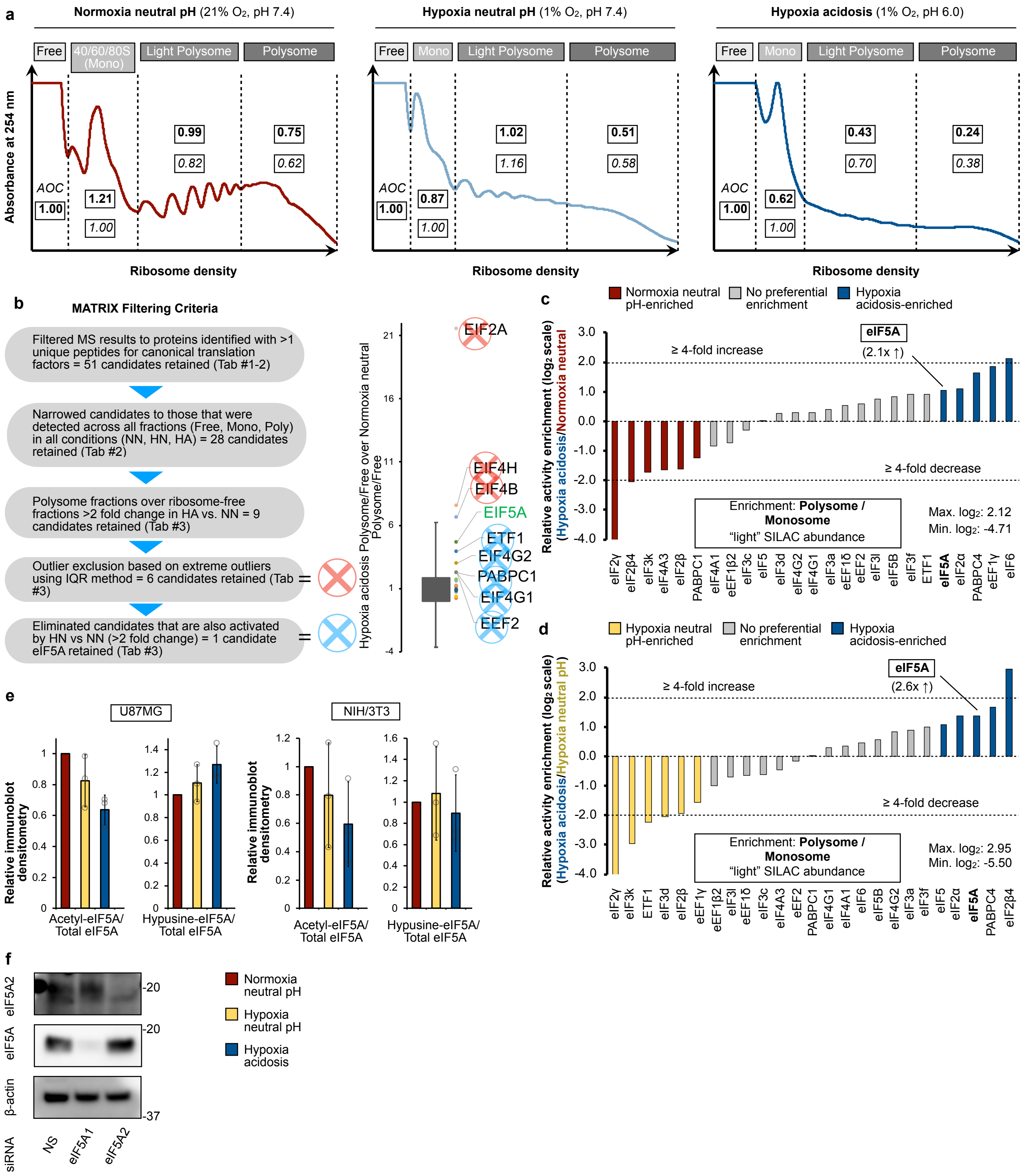


**Supplementary Figure 2**. (a) Ribosome density profiles of U87MG subjected to basal normoxia neutral pH, hypoxia neutral pH, and hypoxia acidosis conditions. AOC: area under curve. Bolded numbers: normalized to free fraction AOC. Regular numbers: normalized to monosome fraction. (b) Filtering criteria for MATRIX analysis in Fig. 1 b-c and Supplementary Fig. 2 b-c, Tab number refers to excel tab in MATRIX_sourcefile. MATRIX analysis of differential translation factor utilization in human cells (U87MG) exposed to hypoxia acidosis (1% $O_2$, pH 6.0, 24 hr) compared to (c) basal (21% $O_2$, pH 7.4, 24 hr) and (d) hypoxia neutral pH (1% $O_2$, pH 7.4, 24 hr) conditions, using the ratio of polysome to monosome abundance as the readout. Hypoxia acidosis-activated translation factors (dark blue bars); Basal-activated translation factors (red bars); hypoxia neutral pH-activated translation factors (light blue bars). (e) Immunoblot densitometry representing mean ± SD (n=3), relative to normoxia neutral pH. (f) Representative immunoblots of U87MG treated with indicated siRNA for 72 hrs showing specificity of reagents used for eIF5A1 silencing throughout manuscript.

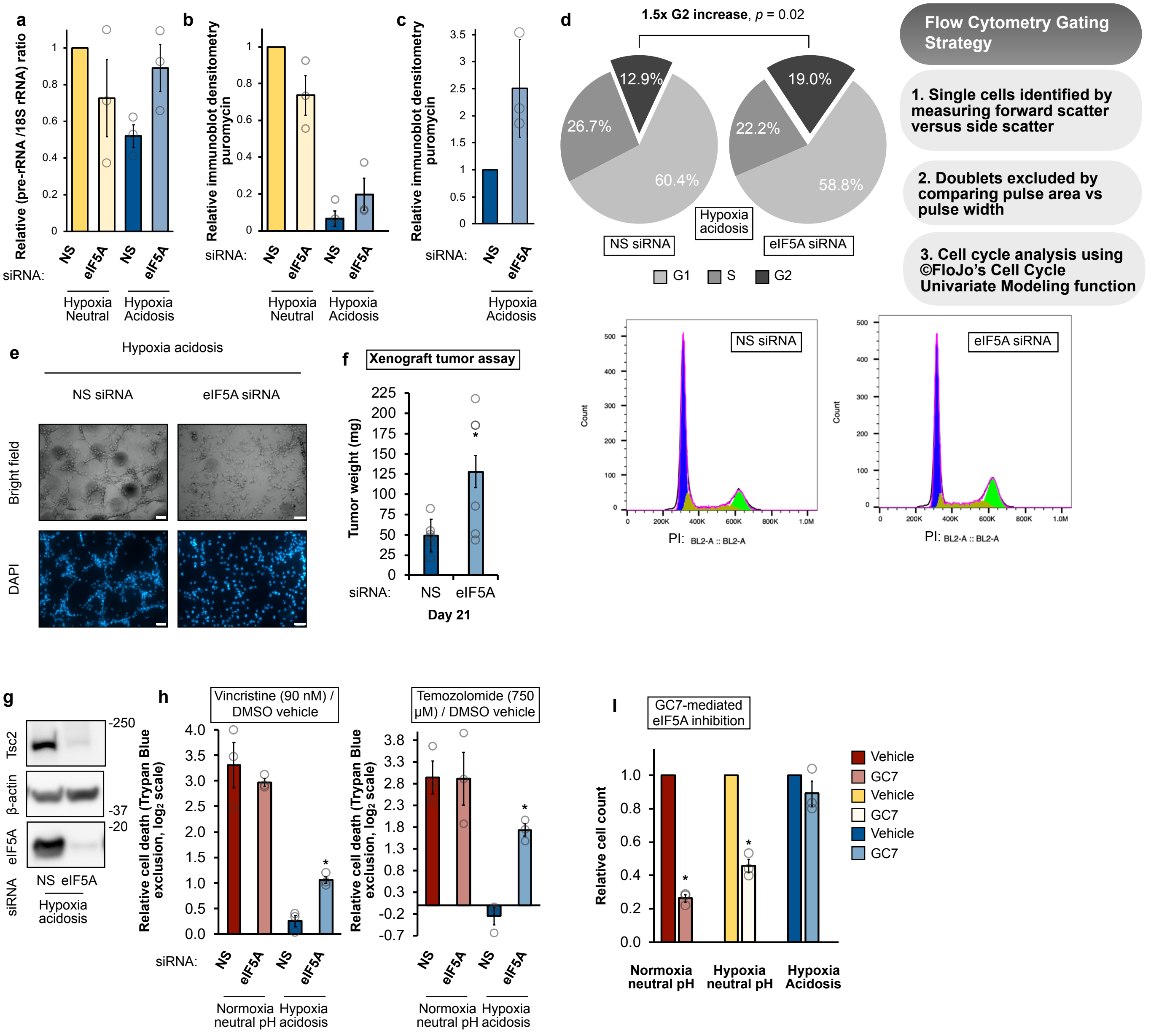


**Supplementary Figure 3**. (a) Transcriptional intensity [Data represents mean ± SEM (n=3)], and (b) translational intensity [Data represents mean ± SD (n=3)] in U87MG replete or depleted of eIF5A under hypoxia acidosis and hypoxia neutral conditions, relative to hypoxia neutral pH. (c) Immunoblot densitometry representing mean ± SD (n=3) (d) Left Top: Cell cycle analysis by flow cytometry, Right top: Gating strategy for flow cytometry, Bottom: Representative experiments U87MG replete or depleted of eIF5A under hypoxia acidosis conditions (n=3). (e) cell morphology and patterning of U87MG replete or depleted of eIF5A under hypoxia acidosis conditions. Scale bars: 100 μm. (n=5), student's two-tail t-test p=.022. (f) Tumor weight measurements of mouse xenograft tumor formation assays using MCF7 replete or depleted of eIF5A and pre-treated with hypoxia acidosis for 48 hr. Data represent mean ± SEM (n=6). * indicates p = 0.036 compared to NS siRNA, two-sided student's t-test. (g) Immunoblot of eIF5A and Tsc2 levels 7 days after siRNA treatment (h) Effect on cell death of eIF5A silencing on U87MG sensitivity to conventional anti-proliferative drugs vincristine and temozolomide under basal or hypoxia acidosis conditions. Data represent mean±SEM (n=3). * indicates p = 0.045, 0.048 (eIF5A siRNA + Vinchrisitne, eIF5A siRNA + Temozolomide) compared to hypoxia acidosis NS siRNA control, student's t-test. (i) Effect of the hypusination inhibitor GC7 on cell number in U87MG subjected to indicated conditions. Data

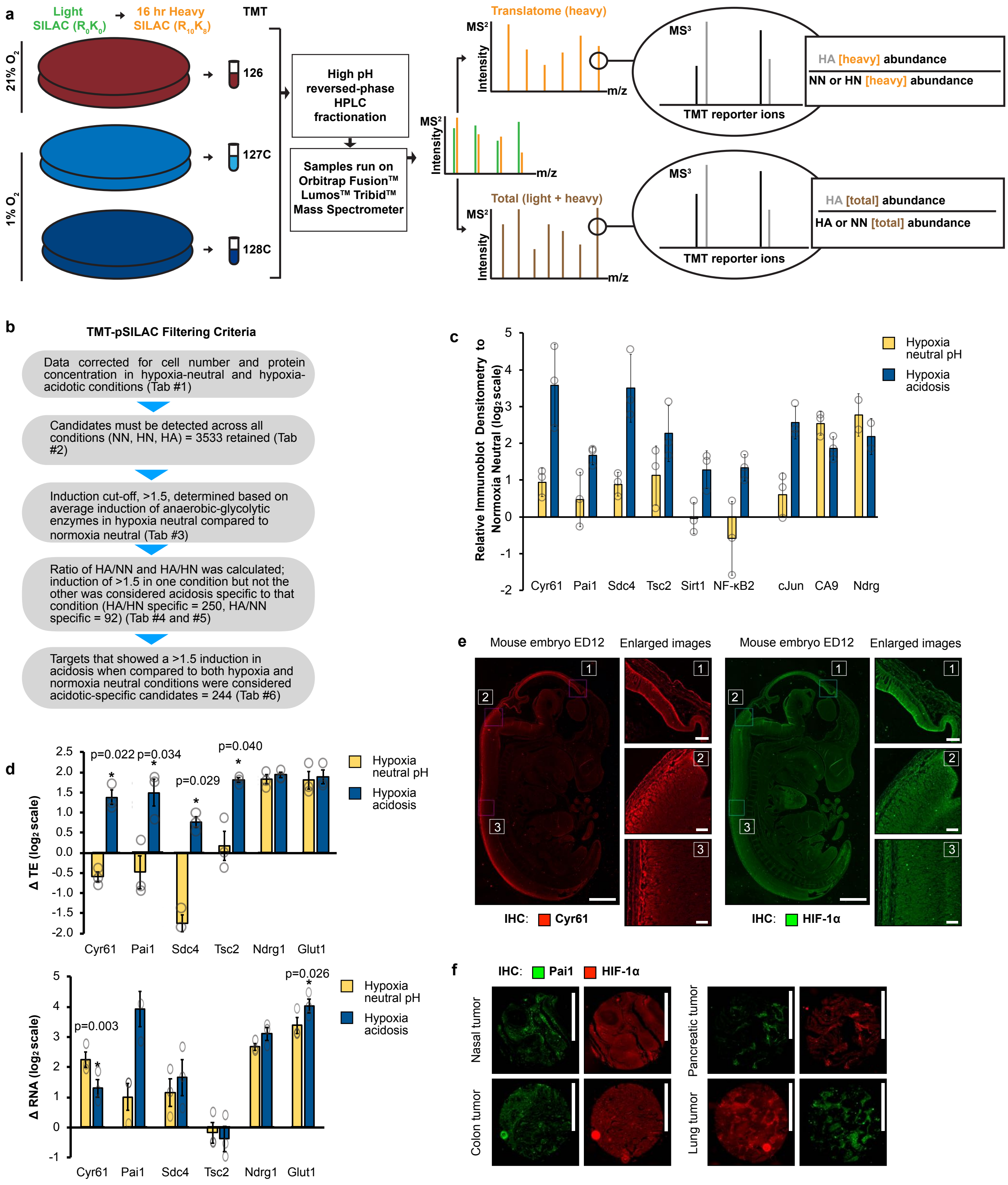


**Supplementary Figure 4.** (a) Workflow schematic of protein output (translatome) analysis by TMT-pSILAC and mass spectrometry. (b) Filtering criteria for TMT-pSILAC analysis in Fig. 3 a-c. Tab number refers to excel tab in TMT-pSILAC_sourcefile. (c) Immunoblot densitometry representing mean ± SD (n=3) (d) qRT-PCR measurements of TE (*top panel*) and steady-state mRNA level (*bottom panel*) changes for hypoxia acidosis-specific translatome targets. Data represent mean ± SEM (n=3), normalized to normoxia neutral pH conditions. * indicates $p < 0.05$ compared to hypoxia neutral pH control (exact p-values in figure), two-sided student's t-test. (e) Individual fluorescence channels for images presented in Figure 4g (n=3)., scale bar in zoomed out image = 1000um, scale bar for zoomed in images = 100 um. (f) Individual fluorescence channels for images presented in Figure 4f (n=3), scale bar = 1000 um..

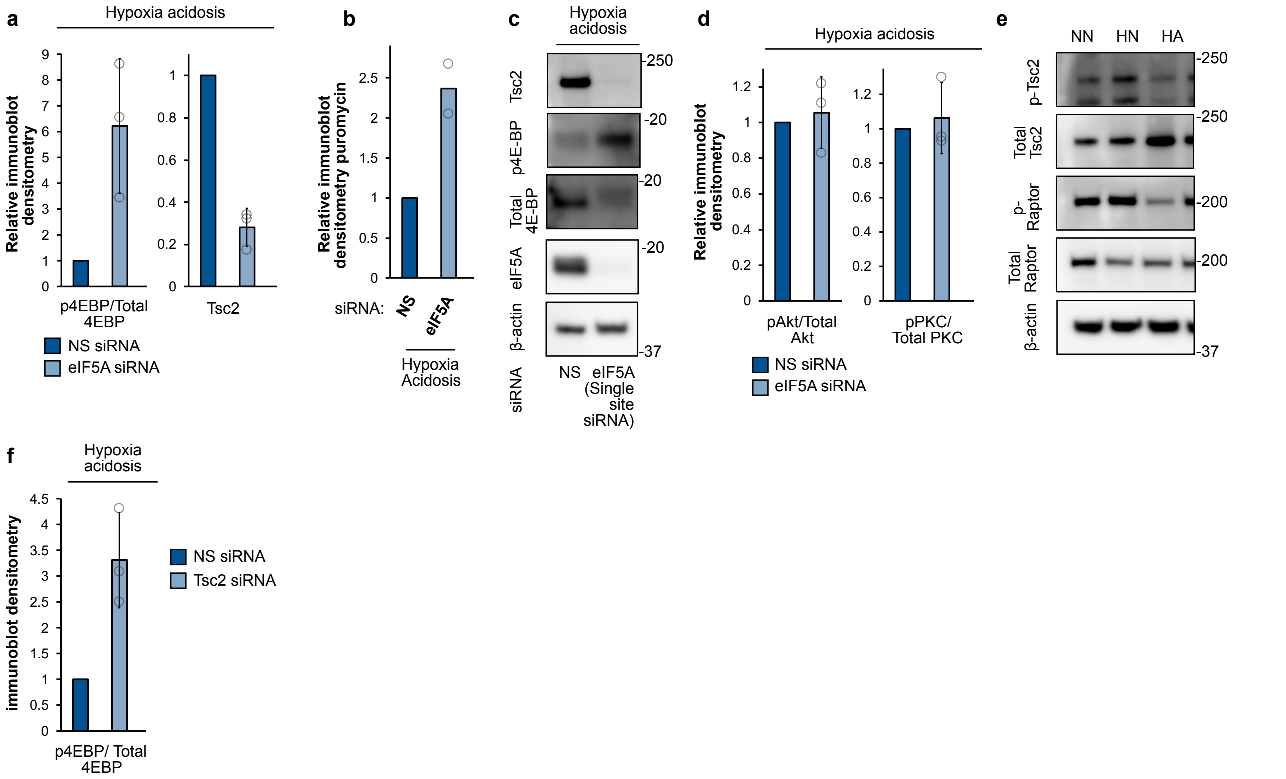


**Supplementary Figure 5**. (a) Densitometry of experiment in Figure 4a. Data represent mean ± SD (n=3), right panel normalized to non-specific siRNA (b) Densitometry of experiment in Figure 4b. Data represent mean (n=2), normalized to non-specific siRNA. (c) Representative immunoblot using eIF5A siRNA targeting one specific site. (d) Densitometry of experiment in Figure 4c. Data represent mean ± SD (n=3) (e) Representative immunoblots of AMP-Kinase targets in NN: normoxia neutral pH; HN: hypoxia neutral pH. (f) Densitometry of experiment in Figure 4d. Data represent mean ± SD (n=3).

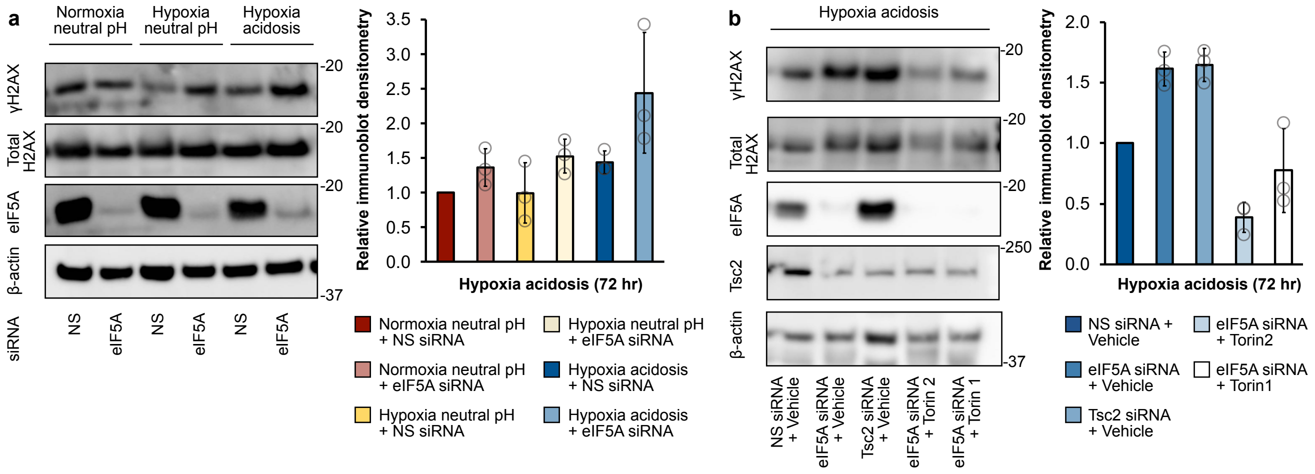


**Supplementary Figure 6**. (a) *Left panel*: Representative immunoblots of U87MG replete of depleted of eIF5A under hypoxia acidosis conditions. *Right panel*: densitometry represent mean ± SD (n=3). (b) *Left panel*: representative immunoblots of U87MG depleted of Tsc2, and in eIF5A-replete or -depleted cells treated with mTORC1 inhibitors (Torin 1 and 2) under hypoxia acidosis conditions. *Right panel*: densitometry represent mean ± SD (n=3).

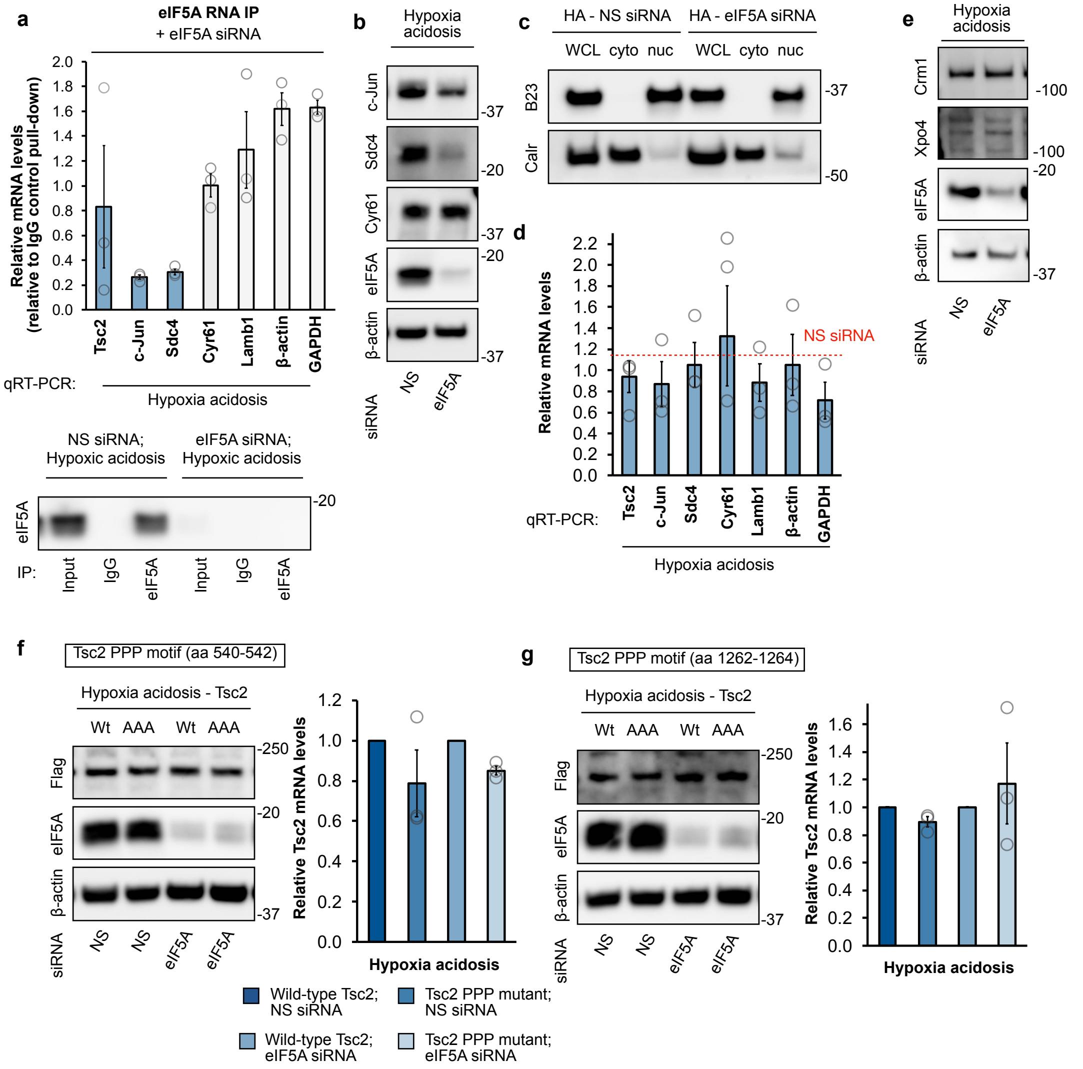


**Supplementary Figure 7**. (a) Control co-IP experiment of Figure 6a in U87MG cells depeleted of eIF5A: mRNA levels of eIF5A regulated and nonregulated mRNAs relative to IgG isotype control pull-down (*top panel*) and representative immunoblot (*bottom panel*) of eIF5A RNA immunoprecipitation experiments. (b) Representative immunoblots of U87MG replete or depleted of eIF5A under hypoxia acidosis conditions (n=3). (c) Control immunoblots for subcellular fraction data presented in Figure 6b. HA: hypoxia acidosis; cyto: cytoplasmic; nuc: nuclear. Calreticulin (calr) and B23 represent markers of the cytosolic and nuclear compartments, respectively (n=3). (d) Effect of eIF5A knockdown on steady-state mRNA levels of indicated targets under hypoxia acidosis conditions. NS: non-silencing. Data represent mean ± SEM (n=3), relative to NS siRNA. (e) Effect of eIF5A knockdown on protein levels of the nuclear transporters Crm1 and Xpo4 under hypoxia acidosis conditions (n=3) (f, g) *Left panel*: representative immunoblots indicating the effect of mutating Tsc2 PPP motifs ((f): aa 540-542; (g): aa 1262-1264) on steady-state protein expression of Flag-tagged Tsc2 in U87MG replete or depleted of eIF5A under hypoxia acidosis conditions. WT: wild-type. AAA: AAA mutant. *Right panel*: qRT-PCR measurements of steady-state Tsc2 mRNA under the same conditions, Data represent mean ± SEM (n=3).

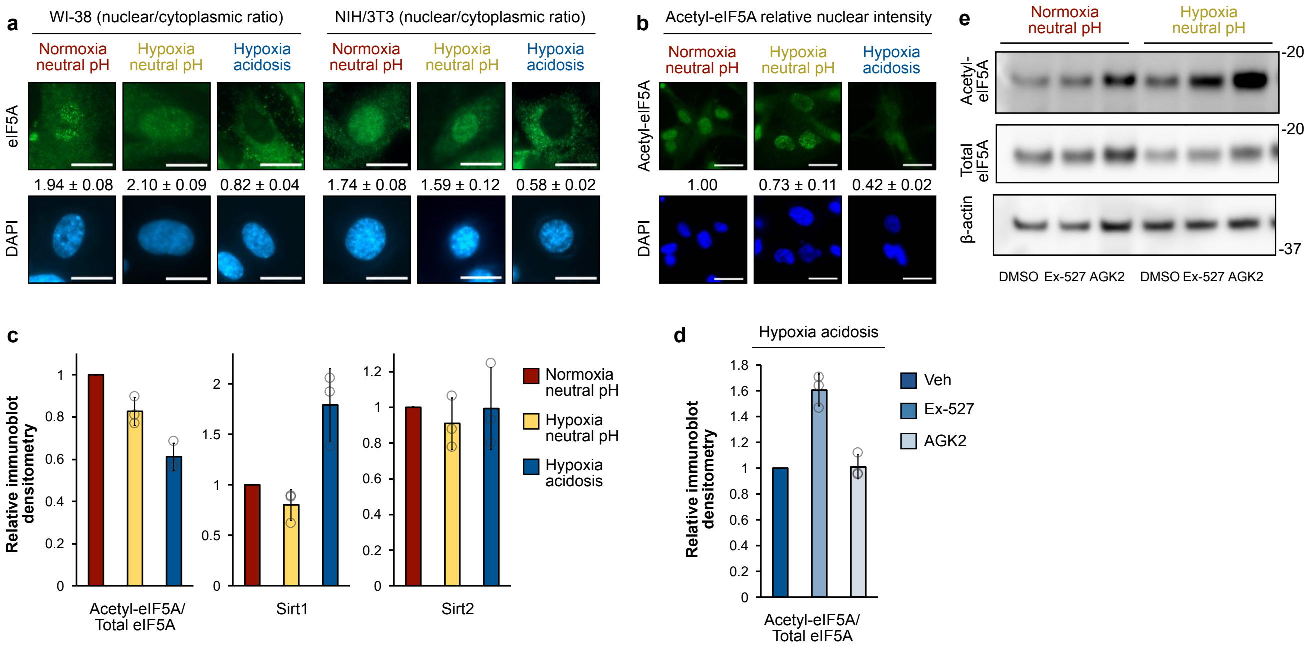


**Supplementary Figure 8**. (a) Representative images of eIF5A immunocytochemistry showing eIF5A subcellular localization in human (WI-38) and mouse (NIH/3T3) cells subjected to indicated conditions. Data represent mean ± SEM (n=10). Scale bars: 20 µm. (b) Representative images of acetyl-eIF5A immunocytochemistry in U87MG subjected to indicated conditions. Data represent mean ± SEM (n=10). Scale bars: 20 µm. (c) Immunoblot densitometry representing mean ± SD (n=3), Sirt1 and Sirt2 measurements relative to normixa neutral pH (d) Immunoblot densitometry of experiment in Figure 7c representing mean ± SD (n=3), relative to veh (e) Representative immunoblots of U87MG treated with the indicated compounds under the indicated conditions. Ex-527: Sirt1 inhibitor; AGK2: Sirt2 inhibitor; DMSO: vehicle (n=2).
