# Supplementary material for: A translational program that suppresses metabolism to shield the genome
Source: Nat Commun. 2020 Nov 13;11:5755. doi: 10.1038/s41467-020-19602-2 (PMC7666154; doi:10.1038/s41467-020-19602-2)
Supplement: Supplementary file 3 — Reporting Summary [file 41467_2020_19602_MOESM3_ESM.pdf]

nature research

Corresponding author(s): Dr. Stephen Lee

Last updated by author(s): 09/26/2020

Reporting Summary

Nature Research wishes to improve the reproducibility of the work that we publish. This form provides structure for consistency and transparency in reporting. For further information on Nature Research policies, see Authors & Referees and the Editorial Policy Checklist.

Statistics

For all statistical analyses, confirm that the following items are present in the figure legend, table legend, main text, or Methods section.

n/a	Confirmed	
☐	☒	The exact sample size (*n*) for each experimental group/condition, given as a discrete number and unit of measurement
☐	☒	A statement on whether measurements were taken from distinct samples or whether the same sample was measured repeatedly
☐	☒	The statistical test(s) used AND whether they are one- or two-sided<br>*Only common tests should be described solely by name; describe more complex techniques in the Methods section.*
☐	☒	A description of all covariates tested
☐	☒	A description of any assumptions or corrections, such as tests of normality and adjustment for multiple comparisons
☐	☒	A full description of the statistical parameters including central tendency (e.g. means) or other basic estimates (e.g. regression coefficient) AND variation (e.g. standard deviation) or associated estimates of uncertainty (e.g. confidence intervals)
☐	☒	For null hypothesis testing, the test statistic (e.g. *F*, *t*, *r*) with confidence intervals, effect sizes, degrees of freedom and *P* value noted<br>*Give P values as exact values whenever suitable.*
☒	☐	For Bayesian analysis, information on the choice of priors and Markov chain Monte Carlo settings
☒	☐	For hierarchical and complex designs, identification of the appropriate level for tests and full reporting of outcomes
☒	☐	Estimates of effect sizes (e.g. Cohen's *d*, Pearson's *r*), indicating how they were calculated

Our web collection on statistics for biologists contains articles on many of the points above.

Software and code

Policy information about availability of computer code

Data collection	Mass spectrometry: Data were collected using an Orbitrap Fusion Lumos Tribrid mass spectrometer (ThermoFisher Scientific) (TMT-pSILAC) or an LTQ-Orbitrap Elite hybrid mass spectrometer (Thermo-Fisher) (MATRIX).
Data analysis	Mass spectrometry: Mass spectrometry: MS raw files were processed using Proteome Discoverer 2.2 (Thermo Fisher Scientific) (TMT-pSILAC). MS raw files were processed using PEAKS software (v8.5, Bioinformatics Solutions Inc.) (MATRIX).<br>Immunoblot densitometry and cytology quantification was performed using ImageJ software (v1.5, NIH).<br>Cell cycle analysis was performed using FloJo v10.

For manuscripts utilizing custom algorithms or software that are central to the research but not yet described in published literature, software must be made available to editors/reviewers. We strongly encourage code deposition in a community repository (e.g. GitHub). See the Nature Research guidelines for submitting code & software for further information.

Data

Policy information about availability of data

All manuscripts must include a data availability statement. This statement should provide the following information, where applicable:

- Accession codes, unique identifiers, or web links for publicly available datasets
- A list of figures that have associated raw data
- A description of any restrictions on data availability

The data that support this study are available from the corresponding author upon reasonable request. Mass spectrometry datasets are available via the ProteomeXchange accessions: PXD006799, PXD015643. The mass spectrometry proteomics data have been deposited to the ProteomeXchange Consortium via the PRIDE111 partner repository with the dataset identifier PXD015643 and 10.6019/PXD015643; and PXD015643 and 10.6019/PXD006799. MS data were searched against the Human Uniprot Database (https://www.uniprot.org) consisting of only reviewed entries using the Sequest HT and MS Amanda 2.0 search engines.. The source data underlying all Figures and Supplementary Figures are provided as a Source Data file including: Figure 1d-f; Figure 2a-h, j; Figure 3d; Figure 4a-k; Figure 5a-e; Figure 6a-b, d, f-g; Figure 7b-c, e-h; Supplementary Figure 1a-f; Supplementary Figure 2e-f; Supplementary Figure 3a-d, g-i; Supplementary Figure 4c-d;

Supplementary Figure 5a-f; Supplementary Figure 6a-b; Supplementary Figure 7a-g; Supplementary Figure 8c-e. Source data for Figure 1b-c and Supplementary Figure 2c-d are provided in MATRIX_source file. Source data for Figure 3a-c are provided in TMT-pSILAC_sourcefile.

Field-specific reporting

Please select the one below that is the best fit for your research. If you are not sure, read the appropriate sections before making your selection.

☒ Life sciences ☐ Behavioural & social sciences ☐ Ecological, evolutionary & environmental sciences

For a reference copy of the document with all sections, see nature.com/documents/nr-reporting-summary-flat.pdf

Life sciences study design

All studies must disclose on these points even when the disclosure is negative.

Sample size	No sample size calculations were performed. We performed a minimum of three independent experiments, which sufficiently confirmed reproducibility, and enabled statistical analyses.
Data exclusions	No data were excluded.
Replication	Experiments were performed at least three independent times, and independent approaches were performed to ensure the reproducibility of experimental findings. Exact number of independent experiments ('n') are provided in figure legends.
Randomization	Randomization was not performed in our studies per se. For each experiment, cell lines and genetically identical organisms used were derived from the same parent population/passage.
Blinding	Blinding was not performed in our studies, as our pre-determined protocols required research personnel to have knowledge of treatment conditions. Objective, quantitative approaches were used to gather and analyze experimental data.

Reporting for specific materials, systems and methods

We require information from authors about some types of materials, experimental systems and methods used in many studies. Here, indicate whether each material, system or method listed is relevant to your study. If you are not sure if a list item applies to your research, read the appropriate section before selecting a response.

Materials & experimental systems

n/a	Involved in the study	
☐	☒	Antibodies
☐	☒	Eukaryotic cell lines
☒	☐	Palaeontology
☐	☒	Animals and other organisms
☒	☐	Human research participants
☒	☐	Clinical data

Methods

n/a	Involved in the study	
☒	☐	ChIP-seq
☐	☒	Flow cytometry
☒	☐	MRI-based neuroimaging

Antibodies

Antibodies used	GLUT1 (Novus Biologicals, # NB110-39113), PAI1 (Proteintech, 13801-1-AP), Puromycin (3RH11) (Kerafast, #EQ0001), β-actin (C4) (Santa Cruz Biotechnology, #sc-47778), EIF5A (abcam, ab32443), EIF3D (Proteintech, # 10219-1-AP), EIF3K (Proteintech, # 10640-1-AP), acetyl-EIF5A (Lys47) (Boster Bio, #P01727), CYR61 (Proteintech, 26689-1-AP), SDC4 (R&D Systems, #P31431), SIRT1 (Proteintech, #13161-1AP), SIRT2 (Proteintech, #19655-1-AP), NFKB2 (Proteintech, #10409-2-AP), 4E-BP (Cell Signaling, #9452S), Phospho-4EBP (Ser65) (Cell Signaling, #9451S), TSC2 (D93F12) (Cell Signaling, #4308S), gamma-H2AX (Novus, NB100-384), H2AX (Abcam, Ab11175), CRM-1 (Novus, NB100-79802), XPO-4 (Abcam, ab133237), eIF5A2 (Thermo, #PA5-30770), Phospho-Raptor (Ser792) (Cell Signaling, #89146S), RAPTOR (Cell Signaling, #2280), Phospho-Tuberin/TSC2 (Ser1387) (Cell Signaling, #5584S), NDRG1 (Abcam, #ab37897), Anti-Hypusine (mAbHpu24) antibody was kindly provided by Genentech, anti-eIF5A (SantaCruz, sc-390202). KI-67 (SantaCruz, sc-23900), P21 (Proteintech, 10355-1-AP), HIF-1 alpha (Novus, #AF1935), CYR61 (GeneTex, N1C3), anti-DIG-Fluorescein antibody (Sigma, 11207750910).
Validation	Target specificity in human samples were verified by immunoblot for the following primary antibodies in our study by RNAi-mediated silencing: EIF5A (abcam, ab32443), TSC2 (D93F12) (Cell Signaling, #4308S), SIRT1 (Proteintech, #13161-1AP), eIF5A2 (Thermo, #PA5-30770). acetyl-EIF5A (Lys47) (Boster Bio, #P01727) was confirmed by immunoblot activity assay using known inhibitors of acetylation (AGK2 and ex527). Translatome targets Cyr61, Pai1, Sdc4, Nfkb2, Tsc2 identified by mass spec were validated by immunoblot [CYR61 (Proteintech, 26689-1-AP), PAI1 (Proteintech, 13801-1-AP), SDC4 (R&D Systems, #P31431), SIRT1 (Proteintech, #13161-1AP), NFKB2 (Proteintech, #10409-2-AP), TSC2 (D93F12) (Cell Signaling, #4308S)] and immunofluorescence [CYR61 (GeneTex, N1C3), PAI1 (Proteintech, 13801-1-AP)]. Phospho-4EBP (Ser65) (Cell Signaling, #9451S) was validated by us using siRNA against Tsc2.

P21 (Proteintech, 10355-1-AP), PAI1 (Proteintech, 13801-1-AP), SIRT1 (Proteintech, #13161-1AP), NFKB2 (Proteintech, #10409-2-AP), EIF3D (Proteintech, # 10219-1-AP) was validated by SDS-immunoblot, IP immunoblot, and immunofluorescent experiments by manufacturer. CYR61 (Proteintech, 26689-1-AP), EIF5A (abcam, ab32443), CRM-1 (Novus, NB100-79802), eIF5A2 (Thermo, #PA5-30770), SDC4 (R&D Systems, #P31431), β-actin (C4) (Santa Cruz Biotechnology, #sc-47778), KI-67 (SantaCruz, sc-23900), anti-eIF5A (SantaCruz, sc-390202), CYR61 (GeneTex, N1C3), were validated by SDS-immunoblot, and immunofluorescent experiments by manufacturer. SIRT2 (Proteintech, #19655-1-AP) was validated by IP immunoblot, and KD immunofluorescent experiments by manufacturer. Acetylated-EIF5A (Lys47) (Boster Bio, P01727), GLUT1 (Novus Biologicals, # NB110-39113), Raptor (Cell Signaling, #2280), XPO-4 (Abcam, ab133237), H2AX (Abcam, Ab11175), puromycin (3RH11) (Kerafast, #EQ0001), NDRG1 (Abcam, #ab37897), EIF3K (Proteintech, #10640-1-AP) were validated by immunoblot by manufacturers. HIF-2α (Novus Biologicals, #NB100-122), HIF-1 alpha (Novus, #AF1935) were validated by immunoblot, overexpression and activity based immunoblot experiments using known inducers and inhibitors, and immunofluorescence by manufacturers. gamma-H2AX (Novus, NB100-384), was validated by immunoblot and experiments inducing DNA damage both immunoblot and immunofluorescence. 4E-BP (Cell Signaling, #9452S), TSC2 (D93F12) (Cell Signaling, #4308S), was validated by IP immunoblot and KD immunoblot by manufacturers. Phospho-4EBP (Ser65) (Cell Signaling, #9451S), Phospho-Raptor (Ser792) (Cell Signaling, #89146S), Phospho-Tuberin/Tsc2 (Ser1387) (Cell Signaling, #5584S), Anti-Hypusine (mAbHpu24) were validated by functional immunoblot experiments by manufacturer. Empirical information on antibody target specificity in human samples for immunoblot applications are available on corresponding manufacturer websites.

Eukaryotic cell lines

Policy information about cell lines

Cell line source(s)	All cell lines (U87MG, MCF7, WI38, PC3, A549, HCT116, NIH/3T3) were purchased from the American Type Culture Collection.
Authentication	Cell lines were authenticated by cytogenetic analysis, STR profiling, and isoenzyme profiling by the manufacturer.
Mycoplasma contamination	All cell lines tested negative for mycoplasma contamination every 6 months.
Commonly misidentified lines (See ICLAC register)	none

Animals and other organisms

Policy information about studies involving animals; ARRIVE guidelines recommended for reporting animal research

Laboratory animals	Xenografts were generated through dorsal cell suspension injection (MCF7, 1.3e6 cells/mouse; U87MG, 5e6 cells/mouse; 50% Matrigel in PBS) in NOD scid gamma (NSG) mice approximately equal number of males and females. Mice were 12-16 weeks old. Tumor volumes were measured by high-frequency ultrasound (Vevo 3100, Visualsonics). After three weeks, mice were euthanized, tumors collected, weighted and processed for histological analysis. Colonies of SCID mice are housed in the Batchelor Building Research Animal Facility, which is fully accredited by the AAALAC and operating under the University of Miami Animal Welfare Assurance, #A-3224-01. Specific pathogen free mice are obtained from Charles River Laboratories, known for high-quality,reliable research animals. The Miller School of Medicine Division of Veterinary Resources assists with daily care and routine cage management.
Wild animals	No wild animals were used
Field-collected samples	No field collected samples were used.
Ethics oversight	University of Miami Institutional Animal Care and Use Committee (IACUC): 18-097-LF

Note that full information on the approval of the study protocol must also be provided in the manuscript.

Flow Cytometry

Plots

Confirm that:

☒ The axis labels state the marker and fluorochrome used (e.g. CD4-FITC).

☒ The axis scales are clearly visible. Include numbers along axes only for bottom left plot of group (a 'group' is an analysis of identical markers).

☒ All plots are contour plots with outliers or pseudocolor plots.

☒ A numerical value for number of cells or percentage (with statistics) is provided.

Methodology

Sample preparation	Cells were harvested in PBS and fixed in 70% EtOH for 30 minutes at 4°C. Cells were washed 2X in PBS and incubated with 100ug/mL of RNAse. Cells were incubated with 200 ul of Propidium Iodide (50 ug/mL) for 30 minutes and cells were processed through flow cytometer

Instrument	Attune NxT Flow Cytometer (Invitrogen)
Software	Attune™ NxT Software - data collection<br>FloJo v10 - data analysis
Cell population abundance	>20,000 cells were analyzed per condtion in every experiment. Determined after gating out doublets and dead cells according to FloJo analysis.
Gating strategy	1. Single cells identified by measuring forward scatter versus side scatter<br>2. Doublets excluded by comparing pulse area vs pulse width<br>3. Cell cycle analysis using ©FloJo's Cell Cycle Univariate Modeling function

☒ Tick this box to confirm that a figure exemplifying the gating strategy is provided in the Supplementary Information.
